# Adsorption of amino acids on MFI-type zeolite: DFT calculations and experimental results

**DOI:** 10.1186/1758-2946-4-S1-P38

**Published:** 2012-05-01

**Authors:** Kai Stueckenschneider, Achim Zielesny, Gerhard Schembecker

**Affiliations:** 1 Department of Biochemical and Chemical Engineering, TU Dortmund, Dortmund, 44227, Germany; 2 Institute for Bioinformatics and Chemoinformatics, University of Applied Sciences Gelsenkirchen, Recklinghausen, 45665, Germany

## 

Adsorption is a common unit operation in separation and purification of biotechnological products where chromatography steps can make up more than half the amount of the total purification costs [1]. The molecular mechanisms of adsorption are still not understood in detail. Further understanding of interactions between adsorbent surfaces and adsorptives could help to facilitate process design in a more cost efficient manner.

In this work the interaction of MFI-type zeolite MFI-27 (Al/Si=13) with Alanine and Phenylalanine is investigated by quantum chemical (QM) calculations which are compared to experimental adsorption data.

For the QM calculations T3-clusters are used as MFI-27 surface models which were shown to be successful e.g. in the case of protolytic cracking of alkanes [2,3]. In order to model different pH-values Alanine and Phenylalanine are applied in their protonated, zwitterionic and deprotonated state. Geometry optimisations and frequency analysis of all molecular structures are performed with Density Functional Theory (DFT) using the B3LYP functional with different basis sets. Calculated complex energies are corrected for BSSE and ZPE. Adsorption isotherms are derived from corresponding experiments.

Regarding the adsorption isotherms it is shown that a high adsorption of Alanine and Phenylalanine on MFI-27 takes place at low pH values (near the pK_a_ of the amino acids). Less adsorption occurs with an increased pH equalling the amino acids’ isoelectric points. At the pH of the amico acids’ pK_b_ values adsorption is no longer observed.

These trends can be correlated with the corresponding QM calculations. High binding energies are calculated for the protonated amino acids. Zwitterionic states lead to lower binding energies. The deprotonated amino acids do not show any binding affinity to MFI-27.

These first results indicate the reliability of the applied methods for our model system.
